# A Test for the Assessment of Pragmatic Abilities and Cognitive Substrates (APACS): Normative Data and Psychometric Properties

**DOI:** 10.3389/fpsyg.2016.00070

**Published:** 2016-02-12

**Authors:** Giorgio Arcara, Valentina Bambini

**Affiliations:** ^1^Department of Neurosciences, University of PaduaPadua, Italy; ^2^Center for Neurocognition, Epistemology and theoretical Syntax, Institute for Advanced Study (IUSS)Pavia, Italy

**Keywords:** pragmatics, neuropragmatics, neuropsychological assessment, figurative language, discourse

## Abstract

The Assessment of Pragmatic Abilities and Cognitive Substrates (APACS) test is a new tool to evaluate pragmatic abilities in clinical populations with acquired communicative deficits, ranging from schizophrenia to neurodegenerative diseases. APACS focuses on two main domains, namely discourse and non-literal language, combining traditional tasks with refined linguistic materials in Italian, in a unified framework inspired by language pragmatics. The test includes six tasks (Interview, Description, Narratives, Figurative Language 1, Humor, Figurative Language 2) and three composite scores (Pragmatic Productions, Pragmatic Comprehension, APACS Total). Psychometric properties and normative data were computed on a sample of 119 healthy participants representative of the general population. The analysis revealed acceptable internal consistency and good test-retest reliability for almost every APACS task, suggesting that items are coherent and performance is consistent over time. Factor analysis supports the validity of the test, revealing two factors possibly related to different facets and substrates of the pragmatic competence. Finally, excellent match between APACS items and scores and the pragmatic constructs measured in the test was evidenced by experts' evaluation of content validity. The performance on APACS showed a general effect of demographic variables, with a negative effect of age and a positive effect of education. The norms were calculated by means of state-of-the-art regression methods. Overall, APACS is a valuable tool for the assessment of pragmatic deficits in verbal communication. The short duration and easiness of administration make the test especially suitable to use in clinical settings. In presenting APACS, we also aim at promoting the inclusion of pragmatics in the assessment practice, as a relevant dimension in defining the patient's cognitive profile, given its vital role for communication and social interaction in daily life. The combined use of APACS with other neuropsychological tests could also improve our understanding of the cognitive substrates of pragmatic abilities and their breakdown.

## Introduction

Pragmatics concerns the interplay of linguistic content, contextual information and general communicative rules in guiding communication (Grice, 1975; Levinson, 1983; Sperber and Wilson, 2005). Typical domains of investigation in pragmatics are those verbal phenomena in which the gap between the literal meaning and the communicative meaning is clearly visible, and in which context plays a major role. Metaphor, irony and non-literal language in general are among those phenomena, as comprehenders are required to integrate contextual information, including belief and intentions, in order to reach the intended meaning. Also aspects of discourse and conversation such as topic maintenance and coherence are often included in the domain of pragmatics, as speakers need to adhere to rules of appropriateness to context in conducting the verbal exchange.

A long tradition which traced back to the early ‘60s identified the right hemisphere as the site of pragmatic abilities in the brain (Joanette et al., 1990). This claim was based on research with different paradigms such as sentence picture matching task for metaphor (Winner and Gardner, 1977) or completion of jokes (Brownell et al., 1983), as well as discourse analysis approaches to the patients’ speech (Joanette and Brownell, 1990). However, it soon became evident that, in addition to right hemisphere brain damaged patients, a large number of clinical populations, while not being aphasic, show similar pragmatic impairments, including patients with schizophrenia, traumatic brain injury and neurodegenerative diseases (Stemmer, 2008; Bambini, 2010; Bambini and Bara, 2012).

The increasing volume of the literature in clinical pragmatics encouraged the development of standardized assessment tools for acquired pragmatic deficits. Tests for English fall into two main categories: structured batteries assessing the comprehension of non-literal language, such as the Right Hemisphere Communication Battery (Gardner and Brownell, 1986) and the Right Hemisphere Language Battery (Bryan, 1995), and tests for evaluating discourse and conversation produced by patients, such as the Pragmatic Protocol (Prutting and Kirchner, 1987) and the Profile of Communicative Appropriateness (Penn, 1985). Similarly, for Italian, both types of approaches were developed. Some tools assess pragmatic abilities with a main focus on non-literal language, among which the *Batteria sul Linguaggio dell'Emisfero Destro* (BLED) (Rinaldi et al., 2004), the Italian version of the *Protocole Montréal d'Évaluation de la Communication* (MEC) (Tavano et al., 2013), and the Assessment Battery for Communication (ABaCo), which expands the evaluation of communicative abilities to non-verbal pragmatics (Angeleri et al., 2012; Bosco et al., 2012). Other methods focus on the analysis of the patient's speech (Marini et al., 2011), based on discourse analysis and pragmatic notions such as are coherence and cohesion, measuring how sentences are connected and integrated in the global narrative context.

Despite increasing evidence of the vulnerability of the pragmatic aspects of communication in a large number of neurological and psychiatric conditions, and despite the existence of evaluation instruments, pragmatic assessment is rarely integrated in the clinical practice. Several reasons motivate this exclusion. First, language assessment usually concentrates on the formal aspects of language, for which a much larger number of standardized tools exist, in order to detect aphasic syndromes. Communicative disruptions at the pragmatic level, although frequently documented and qualitatively reported, are not considered part of the clinical profile and they are often ascribed to cognitive or social cognition deficits. This situation is probably related also to the cognitive substrates of pragmatics, which is known to be associated with a network of different abilities. Among these, Theory of Mind, i.e., the ability to represent another's mental state (Premack and Woodruff, 1978), seems to play a major role, along with executive functions (i.e., working memory, set-shifting, inhibition, planning and flexibility) (McDonald, 2008; Stemmer, 2008). Although the common opinion is that these abilities do not fully account for pragmatic deficit, the cognitive substrates of pragmatics is still considered as a “puzzle” in the neuropsychological literature (Martin and McDonald, 2003; Champagne-Lavau et al., 2007). The second reason playing against the inclusion of pragmatic assessment is that the available pragmatic tests, while offering a fine-grained profile of the patient's communicative skills, are usually too long for clinical settings (90 min on average), and sometimes difficult to administer and score.

In light of this scenario, we aimed at promoting a better consideration of pragmatic aspects in describing the patient's clinical profile. To pursue this aim, we decided to expand the inventory of tools to assess pragmatic abilities, by producing a new test (Assessment of Pragmatic Abilities and Cognitive Substrates, APACS), with the following major innovative characteristics: (i) inclusion of the major domains of impairments as evidenced in the literature on patients, i.e., discourse and non-literal meaning, compacted in a single tool; (ii) careful selection of the materials, combining refined theoretical notions in pragmatics and discourse analysis as well as psycholinguistic variables, and respecting the ecological validity as much as possible; (iii) brevity and easiness of administration. We built the test in Italian, yet encouraging the development of versions in other languages, granted a careful adaptation especially of the non-literal uses, in the perspective of endorsing cross-national sharing of standardized tools and data pooling also for the important domain of social communication.

With respect to (i), our choice fell on discourse and on non-literal language, including figurative expressions (idioms, metaphors, proverbs) and humor, as these are well explored domains in studies on patients, known to be largely impaired in schizophrenia, traumatic brain injured, and neurodegenerative diseases such as fronto-temporal dementia and amyotrophic lateral sclerosis (Brüne and Bodenstein, 2005; Ash et al., 2014; Marini et al., 2014; Clark et al., 2015). Although pragmatic impairment might affect also other pragmatic dimensions, we believe that discourse and non-literal language might represent two appropriate test-grounds to detect a global deficit in social communication. APACS has the advantage of combining discourse and non-literal language in a single tool while preserving the brevity of the instrument, thus overcoming the traditional separation between tests assessing discourse and tests assessing figurative language^1^. Importantly, studies and meta-analyses in neuropragmatics showed that the comprehension of metaphor, humor, as well as discourse rely on a common extended language network (Ferstl, 2010), extending to Theory of Mind and executive functions hubs, with differences depending on the specific task. The rationale behind APACS acknowledges that pragmatics, while globally depending on context, is not monolithic and different pragmatic aspects might involve different cognitive skills. APACS might indeed be useful also to shed light into the cognitive substrates of pragmatic abilities, which might not completely overlap across tasks and might be differently compromised across pathologies (Champagne-Lavau et al., 2007).

With respect to (ii), great attention was devoted to the construction of the materials. As a general trend, we tried to enhance the realistic nature of the stimuli, by using photographs instead of line drawings, and everyday language as in news articles. Theoretically, we took into account notions from linguistic and pragmatics (e.g., the distinctions among figurative language types such as idioms and metaphors, often blended together in previous tests). Psycholinguistic variables such as familiarity (for figurative expressions) and readability (for narrative texts) were also balanced. For figurative language in particular, research in psycholinguistics showed the importance of familiarity and previous exposure in shaping processing load and mechanisms (Cardillo et al., 2010). When possible, stimuli in APACS were extracted from norms or rating studies collected on the Italian populations, thus balancing the conventionality of the expressions. Other materials in APACS were *ex novo* built paying attention to contextual appropriateness.

With respect to (iii), we employed widely used tasks such as sentence matching or semi-structured interview, so that no special training is required on the clinician's side, thus increasing the easiness of administration. Training requirement is minimal also for the scoring, which in APACS is done on-line based on clear instructions. Administration time averages 35–40 min, depending on the individual's characteristics. As an important caveat, APACS tasks focus on verbal pragmatic abilities as they are used in social communication, but does not directly manipulate contextual settings, neither involve role playing, since the use of these approaches is still controversial (Crockford and Lesser, 1994). To this respect, recent literature is orienting toward the use of functional communication scales as the best measure of communicative skills in social situations, and their impact on functioning (Long et al., 2008).

The final structure of the APACS test includes 6 tasks (Interview, Description, Narratives, Figurative Language 1, Humor, Figurative Language 2) and allows to derive three composite scores (Pragmatic Production, Pragmatic Comprehension, APACS Total). It is advisable to accompany the test in parallel with a neuropsychological assessment, evaluating especially executive functions and social cognition, to unravel different involvement across pragmatic tasks. The full name of the test (“Assessment of Pragmatic Abilities and Cognitive Substrates”) captures this perspective. The use of tests assessing formal aspects of language is also advisable, to dissociate aphasic from “apragmatic” profiles. In what follows we first present the structure of the APACS test, and then we describe the psychometric properties and provide normative data from an Italian population sample.

## Methods

### Stimuli and structure of the APACS test

**Figure 1:**
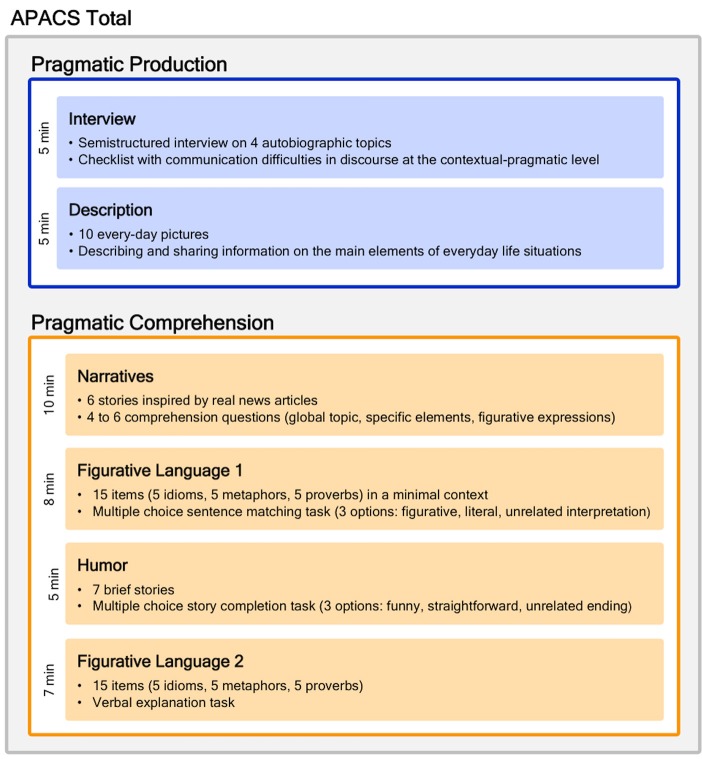
**Structure of the APACS test and derived scores**. The figure shows the six tasks included in the APACS test, and the composite scores derived for Pragmatic Production (light blue background) and Pragmatic Comprehension (light orange background). The APACS Total score (gray background) includes both Pragmatic Production and Pragmatic Comprehension.

The APACS test focuses on the assessment of two main pragmatic domains, namely discourse and non-literal language. The test is divided in two main sections, one devoted to assess production and the other devoted to assess comprehension, for a total of 6 tasks. Three composite scores are derived from the tasks. Below we provide a short description of the six tasks and the three composite scores. Figure 1 summarizes the structure of the test and the derived scores. Examples of items are provided in Supplemental Data Sheet 1: APACS-Item Examples. Further information on APACS can be obtained from the authors.

#### Interview

This task (duration: approximately 5 min) aims at assessing the ability of engaging in conversation though a semi-structured interview, organized around four autobiographical topics: family, home, work, organization of the day, known to be suitable topic to enhance speech in patients (Borovsky et al., 2007). The discourse produced by the subject is assessed according to a checklist including the main parameters of discourse analysis, based on previous approaches to pathological speech (Prutting and Kirchner, 1987; Marini et al., 2011). Several dimensions of discourse are rated on line for the presence of communication difficulties at the contextual-pragmatic level, namely speech (e.g., repetition, incomplete utterances, echolalia), informativeness (over- or under-informativeness, loss of verbal initiative) and information flow (missing referents, wrong order of the discourse elements, abrupt topic shift). Although the focus of the assessment is on verbal pragmatics, the paralinguistic dimension of discourse is included in the rating (e.g., altered intonation, loss of eye-contact, fixed facial expression, abuse of gesture). Also errors in grammar and vocabulary are annotated, based on classic aphasic symptoms such as anomia and paraphasia (Semenza, 2002), as they impact on the communicative effectiveness of the discourse. The frequency of each type of communication difficulty is annotated (always/sometimes/never) and then converted into scores (0/1/2). Maximal score: 44.

#### Description

This task (duration: approximately 5 min) aims at assessing the ability of producing informative descriptions and sharing information of everyday life situations. Compared to the Interview task, here expressive abilities are measured through a more structured task, similar to traditional picture description task, but with higher ecological validity. Ten photographs that depict scenes of everyday life (e.g., a woman waiting at the bus station, a man buying a newspaper in a shop) are presented one by one. The subject is asked to describe the photograph in relation to the main elements that characterize the scene (the location, i.e., the so-called “scene setting topic,” the agent(s) and the action performed by the agent(s)). For each salient element in each picture, a score is assigned differentiating missed identification, partially correct identification, correct identification (0/1/2). Maximal score: 48.

#### Narratives

This task (duration: approximately 10 min) aims at assessing the ability to comprehend discourse and the main aspects of a narrative text. Six stories were built, inspired by real news articles, with increasing length (number of sentences ranging from 4 to 8), and complexity set on a medium difficulty level for subjects with 8 years of schooling, scoring on average 58.5 on the Gulpease readability index (range 0–100) (Lucisano and Piemontese, 1988). Each story includes two non-literal expressions. Stories are read to the subject at normal rate. Following each story, several question items are administered:

- an open question about the global topic of the story, rated 1 when correctly answered or 0;
- 2–4 yes/no questions on specific elements of the story, either main or detail, either stated or implied, as in previous story comprehension tasks (Ferstl et al., 2005), rated 1 when correctly answered or 0;
- 2 questions requiring a verbal explanation relative to the 2 non-literal expressions embedded in the story, rated 2, 1, or 0, based on the accuracy of the explanation, as described below for Figurative Language 2. Maximal score: 56.

#### Figurative language 1

This task (duration: approximately 8 min) aims at assessing the ability to infer non-literal meaning through multiple choice questions, similarly to existing tests (Rinaldi et al., 2004). Fifteen sentences are presented, selected from available databases, with different degrees of lexicalization, including: five highly familiar idioms, average familiarity 6.36 on a 7 point scale, based on existing norms (Tabossi et al., 2011); five novel metaphors, average familiarity 3.78 on a 5 point scale, based on existing ratings (Bambini et al., 2013); five common proverbs extracted from a dictionary of Italian proverbs (Guazzotti and Oddera, 2006). All sentences are provided with a minimal context. For each sentence, three possible interpretations are presented and the subject is asked to choose the one that correctly expresses the figurative meaning. Options include one correct, figurative, interpretation, and two incorrect interpretations, one literal and one unrelated with respect to the target word. Each item is scored either 1 or 0 according to the accuracy. Maximal score: 15.

#### Humor

This task (duration: approximately 5 min) aims at assessing the ability to comprehend verbal humor through multiple choice questions, inspired by the Joke and Story Completion Test (Brownell et al., 1983). The materials consist of seven items, each presenting a brief story. For each story, three possible endings are provided, including: a correct funny ending; an incorrect straightforward non-funny ending; an incorrect unrelated non-sequitur ending. Correct funny endings either play with literal and polysemous meanings, or require to derive non-explicit, unexpected scenarios (Yus, 2008). The subject is asked to select the ending that best functions as the punchline of the story. Each item is scored either 1 or 0 according to the accuracy. Maximal score: 7.

#### Figurative language 2

This task (duration: approximately 7 min) aims at assessing the ability to infer non-literal meanings through verbal explanation, similar to previous tests (Papagno et al., 1995; Amanzio et al., 2008). The materials were selected as for the Figurative Language 1 task and consist of 15 sentences, including 5 highly familiar idioms (average familiarity 6.52), 5 novel metaphors (average familiarity 3.88), and five common proverbs listed in the dictionary. The subject is asked to explain the meaning of each expression. Responses score 2 when the subject provides a good description of the actual meaning of the figurative expression, 1 when the subject provides incomplete explanation, such as concrete examples, but fails in providing a general meaning, 0 when the subject paraphrases the figurative expression, provides a literal explanation, or ignores the expression. Maximal score: 30.

#### Composite scores

Three composite pragmatic scores are computed from the tasks' scores. The Pragmatic Production composite score is calculated from Interview and Description tasks, whereas the Pragmatic Comprehension composite score is calculated from Narratives, Figurative Language 1, Humor and Figurative Language 2 tasks. Each composite score is obtained transforming the original tasks' scores in proportions, and averaging these proportions. Hence, each task contributes with equal weight to the final composite score, which ranges from 0 to 1. Furthermore, the Total APACS score is derived as the mean of the Pragmatic Production and the Pragmatic Comprehension scores. The APACS composite scores allow to coarsely categorize the pragmatic performance of the individuals and can be used to classify patients according to general notions of pragmatic abilities or to easily describe the overall status of pragmatic impairment for clinical purposes.

### Participants

Normative data for APACS were collected from 119 healthy participants. The sample selection was stratified by age and years of education to reflect as much as possible the demographic characteristics of the Italian population. Mean age was 50.03 years (*SD* = 16.79, range 19–89) and mean education was 13.49 years (*SD* = 4.54, range = 5–23). Sixty-five participants were female and 54 were male. Among the participants, 114 were right-handed and 5 were left-handed. Details on the distribution of participants' demographic variables are reported in Table 1. All participants were native speakers of Italian, autonomous in their daily living and had no relevant pathologies that could affect the cognitive performance. Moreover, no participant reported any developmental learning disorder. All participants took part to the study on a voluntary basis and gave their informed consent according to the Helsinki Declaration.

**Table 1:** **Distribution of Age, Education, and Gender for the 119 healthy participants of APACS normative data**.

	**Age**							
**Education**	**19–30**	**31–40**	**41–50**	**51–60**	**61–70**	**71–80**	**80–89**	**Tot M/F**
5–7	0/0	0/0	0/0	0/2	1/4	1/2	1/0	3/8
8–12	0/0	3/0	2/6	4/4	1/1	0/0	0/1	10/12
13–17	4/2	4/2	0/4	11/14	3/4	1/2	1/1	24/29
18–23	8/7	2/3	3/0	2/4	2/1	0/0	0/1	17/16
Tot M/F	12/9	9/5	5/10	17/24	7/10	2/4	2/3	54/65

*The values in each cell indicate the number of males/females*.

### Procedure

The APACS test was administered to each participant in a single session of approximately 35–40 min. Since the APACS test is meant for use on clinical populations, the tasks were presented in a fixed order, as is standard in clinical practice. The order was fixed starting with Interview, as the most natural task in the test situation, and then alternating tasks of different processing load, as follows: *Interview, Description, Narratives, Figurative Language 1, Humor, Figurative Language 2*. Data collection was performed by trained psychologists or linguists. All statistical analyses were performed by means of the free statistical software R (R Core Team, 2015).

## Results

Raw results on APACS for the 119 controls are reported in Table 2. To facilitate the inspection of age and education stratification on APACS scores, results were divided in two age bins (age < 55 years and age ≥ 55 years) and two education bins (education ≤ 13 and education > 13). Results show that healthy controls have very high scores in all age and education bins (see Supplementary Tables 1 in Supplemental Data Sheet 2: APACS-Data Tables and Cut-offs). This makes APACS particularly suited to detect impairments rather than to measure proficiency in healthy individuals.

**Table 2:** **Descriptive statistics of APACS results**.

**Task or composite score**	**Mean**	***SD***	**Median**	**Min**	**Max**	**Kurtosis**	**Skewness**	**Q1**	**Q3**
Interview	43.46	1.20	44	39	44	5.10	−2.40	43.5	44
Description	47.57	1.27	48	43	48	5.53	−2.64	48	48
Narratives	53.40	1.90	54	46	56	4.52	−1.87	53	55
Figurative Language 1	14.77	0.55	15	13	15	3.76	−2.20	15	15
Humor	6.51	0.70	7	5	7	−0.30	−1.04	6	7
Figurative Language 2	27.69	3.32	28	16	30	4.04	−2.10	27.5	30
Pragmatic Production	0.99	0.02	1	0.9	1	4.45	−2.07	0.98	1
Pragmatic Comprehension	0.93	0.08	0.95	0.52	1	6.98	−2.33	0.92	0.98
APACS Total	0.96	0.04	0.97	0.71	1	8.74	−2.51	0.95	0.99

*The table reports the means, standard deviations, median, minimum, maximum, kurtosis, skewness, first quartile and third quartile for APACS scores in the normative data group of 119 participants*.

### Internal consistency

The Internal consistency of APACS was calculated by means of Cronbach's alpha on all items in each APACS task on the whole sample of 119 participants^2^. In particular, we adopted the standardized alpha, based upon the correlations. Results indicate that all APACS tasks have acceptable internal consistency, with alpha values ranging from 0.60 to 0.70. Specifically, the following values were obtained: 0.63 for Interview; 0.65 for Description; 0.66 for Narratives; 0.60 for Figurative Language 1; 0.63 for Humor; 0.70 for Figurative Language 2.

### Test-retest reliability and practice effect

The Test-Retest reliability of APACS was assessed in a subset of 19 participants (mean age = 42.00, *SD* = 14.85; mean education 16.89, *SD* = 4.12) tested at two separate times with a 2-week interval, by the same examiner. A small Test-Retest interval was chosen in order to maximize the possibility to detect undesired practice effects. Results indicate that Test-Retest reliability, calculated by means of Pearson correlations, is good to excellent for all APACS tasks except for Narratives, which showed a remarkably low value (i.e., 0.19, see Table 3). Probably the reason of this low value is the almost ceiling performance of the participants who underwent the Test-Retest combined with the practice effect (see below). Low Test-Retest reliability in the normative sample of neuropsychological tests are not surprising (see for example Spinnler and Tognoni, 1987), especially when a ceiling effect is observed^3^.

**Table 3:** **Test-Retest reliability and practice effect of APACS**.

**Task or composite score**	**Test-Retest reliability**	**Score difference (Retest minus Test)**	***t*-test (Test vs. Retest)**
Interview	0.84	+0.16	*t*_(18)_ = −1.84, *p* = 0.08
Description	0.91	−0.26	*t*_(18)_ = 1.05, *p* = 0.31
Narratives	0.19	+1.47	*t*_(18)_ = −3.29, *p* = 0.004^*^
Figurative Language 1	0.94	+0.05	*t*_(18)_ = −1, *p* = 0.33
Humor	0.74	0	*t*_(18)_ = 0, *p* = 1
Figurative Language 2	0.86	−0.26	*t*_(18)_ = 1.23, *p* = 0.24
Pragmatic Production	0.91	−0.001	*t*_(18)_ = 0.36, 0.72
Pragmatic Comprehension	0.82	−0.005	*t*_(18)_ = −1.21, *p* = 0.24
APACS Total	0.88	0.002	*t*_(18)_ = −0.85, *p* = 0.41

Test-Retest analyses were conducted on a subsample of 19 participants. The following information is provided: task or score name (first column); Test-Retest reliability measured by means of Pearson correlations (second column); practice effect, calculated as the mean difference between the measurements at test and retest (third column); results of the paired t-tests comparing the scores at Test and Retest, with stars (

* *) denoting a significant difference and a potentially harmful practice effect (fourth column)*.

The presence of practice effects in the APACS tasks and composite scores was evaluated by means of a series of paired *t*-tests comparing the scores at the two measurements. A significant practice effect was found only in Narratives, where participants scored slightly better in the second measurement than in the first. All other tasks and composite scores showed no trend of practice effect (see Table 3).

Furthermore, to allow the utilization of APACS for detecting changes over time (for example after a treatment), we employed a statistical method that, given two scores from the same individual, determines if a significant change occurred. Among the many possibilities to define a significant change (Jacobson and Truax, 1991; Collie et al., 2002), we used a regression-based approach (Crawford and Garthwaite, 2006). According to this method, a score in the second measurement is predicted from the score observed in the first measurement. If the score observed at second measurement is far from the predicted value, then a significant change is inferred. The main advantage of using a regression-method is that it takes into account test-retest reliability and factors out both the practice effect and the “regression to the mean” bias (Crawford and Howell, 1998a). Specifically, the method from Crawford and Garthwaite (2006), unlike several other methods, takes into account the fact that the data used to build the regression models derive from a sample drawn from a wider population. For this reason, results derived through regression-based methods are very robust and methodologically they are the gold standard to identify significant changes. Thresholds for significant changes are provided in the Supplementary Tables 2 in Supplemental Data Sheet 2: APACS-Data Tables and Cut-offs.

### Factorial structure and construct validity

The factorial structure of APACS was inspected to study the relationship between APACS task scores. APACS includes different pragmatic domains possibly associated to different cognitive substrates. For this reason, we did not expect that a single factor could explain the variability observed in APACS tasks. Rather, we expected a factorial structure where several domains correlate with the task scores, possibly in relation to the involvement of different cognitive functions.

We performed an exploratory factorial analysis (using a solution with varimax rotation) on all APACS tasks excluding *Description*. This task was excluded because of its almost ceiling distribution of the scores, which made it unsuitable for factorial analysis. A two factors solution provided a satisfactory fit of the data [χ_(1)_ = 0.33, *p* = 0.57]. The correlation between the APACS tasks is reported in Table 4, and the results of the factor analysis are reported in Table 5.

**Table 4:** **Correlations between APACS task scores**.

	**Interview**	**Description**	**Narratives**	**Figurative Language 1**	**Humor**	**Figurative Language 2**
Interview	−					
Description	0.06	−				
Narratives	0.28	0.36^*^	−			
Figurative Language 1	0.26	0.13	0.35^*^	−		
Humor	0.22	0.38^*^	0.60^*^	0.40^*^	−	
Figurative Language 2	0.45^*^	0.08	0.59^*^	0.52^*^	0.49^*^	−

The table reports the Pearson correlations between APACS task scores, performed on the sample of 119 participants. Stars (

* *) denote significant correlations*.

**Table 5:** **Results of factor analysis on APACS tasks**.

**Task**	**Factor 1–loadings**	**Factor 2–loadings**
Interview	0.43	0.13
Narratives	0.48	0.50
Figurative Language 1	0.46	0.30
Humor	0.24	0.97
Figurative Language 2	0.96	0.27

*The table reports the factor loadings for the APACS task, after a factor analysis with varimax rotation. The Description task was not included in the factor analysis due to a marked ceiling effect*.

The inspection of loadings reveals that the first factor is presumably associated with the comprehension of figurative meanings, being mostly correlated to *Figurative Language 1, Figurative Language 2*, and *Narratives* (which includes questions on figurative language). For the second factor, the highest loadings are in Humor and Narratives. Overall, the results from this factor analysis may be taken as evidence that supports construct validity of APACS, as a test able to capture different aspects of the pragmatic competence, possibly related to different cognitive substrates.

### Content validity

Content validity refers to the extent to which the items in a test are appropriate to measure the construct that the test intends to measure. To assess content validity we followed the method adopted in Sacco et al. (2008), by asking five experts in linguistics (4 Linguists and 1 Psycholinguist) to rate on a 5-point Likert scale how each task or score of the APACS test measures the construct it intends to measure. A set of statements was presented to the raters, one for each item or composite score of APACS. For example, for Figurative Language 1, the statement associated to each item was “This item evaluates the ability to understand figurative language.” A score of 1 in the Likert scale indicated “I completely disagree with the statement,” whereas a score of 5 indicated “I completely agree with the statement.” Intermediate value of 3 indicated “I don't agree neither disagree with this statement.” Responses for all items were collapsed within and across judges, to obtain a mean value and a standard deviation for each task. A series of question on the quality of APACS composite scores (Pragmatic comprehension, Pragmatic production, and APACS Total) was also added. The overall mean responses (reported in Table 6) are very high (all above 4.5), indicating that all experts judged that the items of each task and the composite scores were appropriate.

**Table 6:** **Content validity of APACS**.

**Task or composite score**	**Appropriateness**
Interview	4.41 (0.73)
Description	4.71 (0.20)
Narratives	4.81 (0.06)
Figurative Language 1	4.89 (0.08)
Humor	4.86 (0.20)
Figurative Language 2	4.83 (0.15)
Pragmatic Production	4.80 (0.45)
Pragmatic Comprehension	5.00 (0.00)
APACS Total	5.00 (0.00)

*The table shows the content validity of each task and composite score, operationalized as the appropriateness of each item (or score) in assessing the construct measured by the task, evaluated on a 5-point Likert scale. The cells report average values (standard deviations enclosed in round brackets)*.

### Effect of demographic variables on APACS tasks and composite scores

In order to better characterize the effect of age, gender and education on APACS, we performed a series of multiple regressions with each APACS task and composite score as dependent variable. Age and education were included in the regression models as continuous predictors, whereas Gender was included as a factor with two levels (male, female).

For each regression, we used the following regression modeling strategy: starting from an initial model including the three predictors (age, education, and gender) we used a backward elimination of terms, with a method based on Akaike Information Criterion, using the *step* function of R (R Core Team, 2015). After this first term selection, we further removed the terms whose coefficients were not statistically significant. After this procedure of variable selection, the final model on each dependent variable included only significant predictors. We graphically inspected the partial residuals of each variable in each model to investigate if relaxing the assumption of linearity could improve the fit. For all the variables that showed a non-linear trend, we tested if adding quadratic terms yielded to better models. According to the standard regression procedure, if a quadratic term was significant, we kept also the linear term in the model, regardless of its significance.

**Figure 2:**
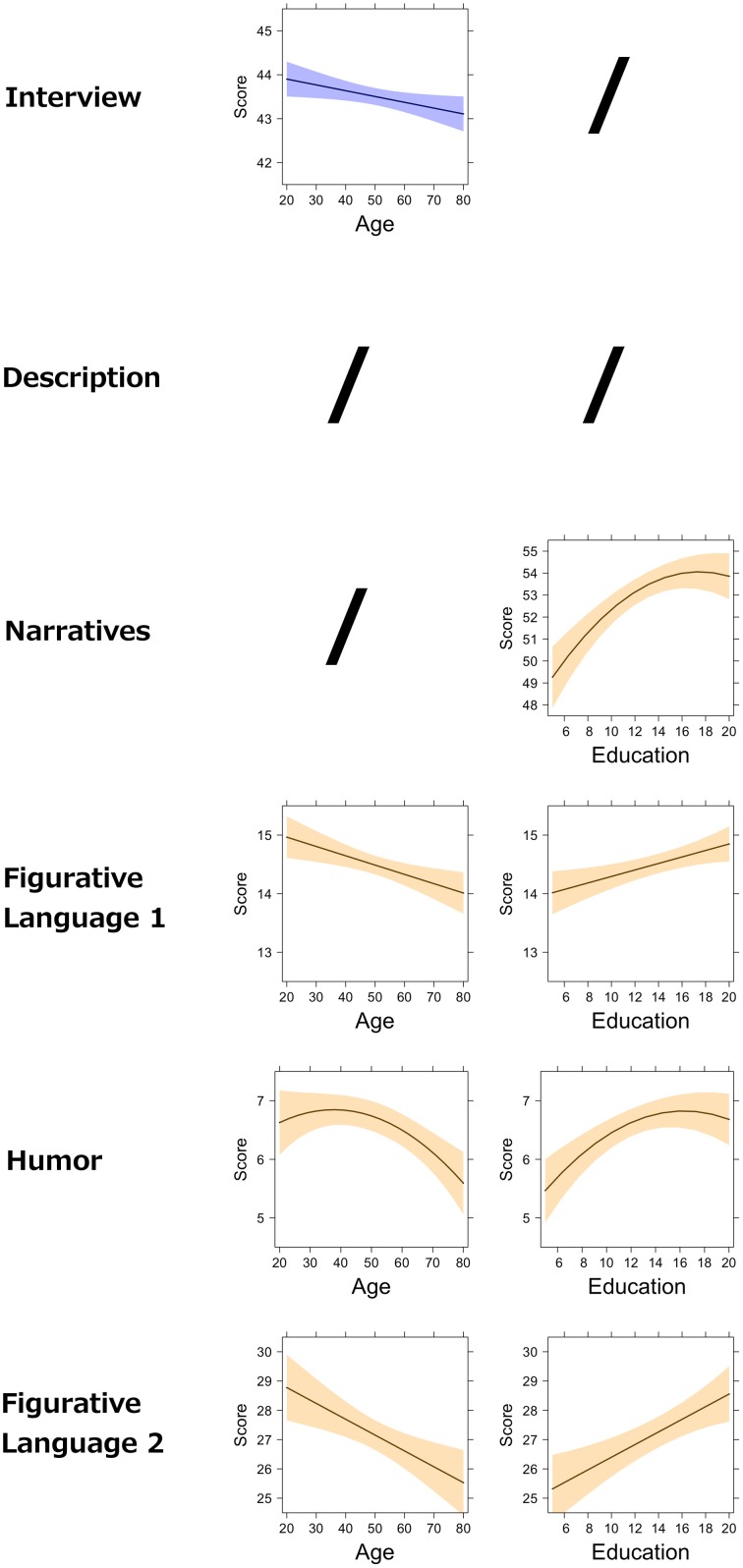
**Effect of demographic variables on APACS tasks**. The figure shows the partial effects of age and education on APACS tasks, as estimated by regression analysis. The figure is an array displaying the APACS tasks (first column) and the effect of age (second column) and education (third column). A slash (“/”) indicates that the effect was not significant in the regression analysis. The black line in each plot represents the predicted score at the APACS task. The colored bands around the line represent point-wise confidence bands around the prediction. Light blue is used for the tasks that compose the Pragmatic Production score. Light orange is used for the tasks that compose the Pragmatic Comprehension score.

**Figure 3:**
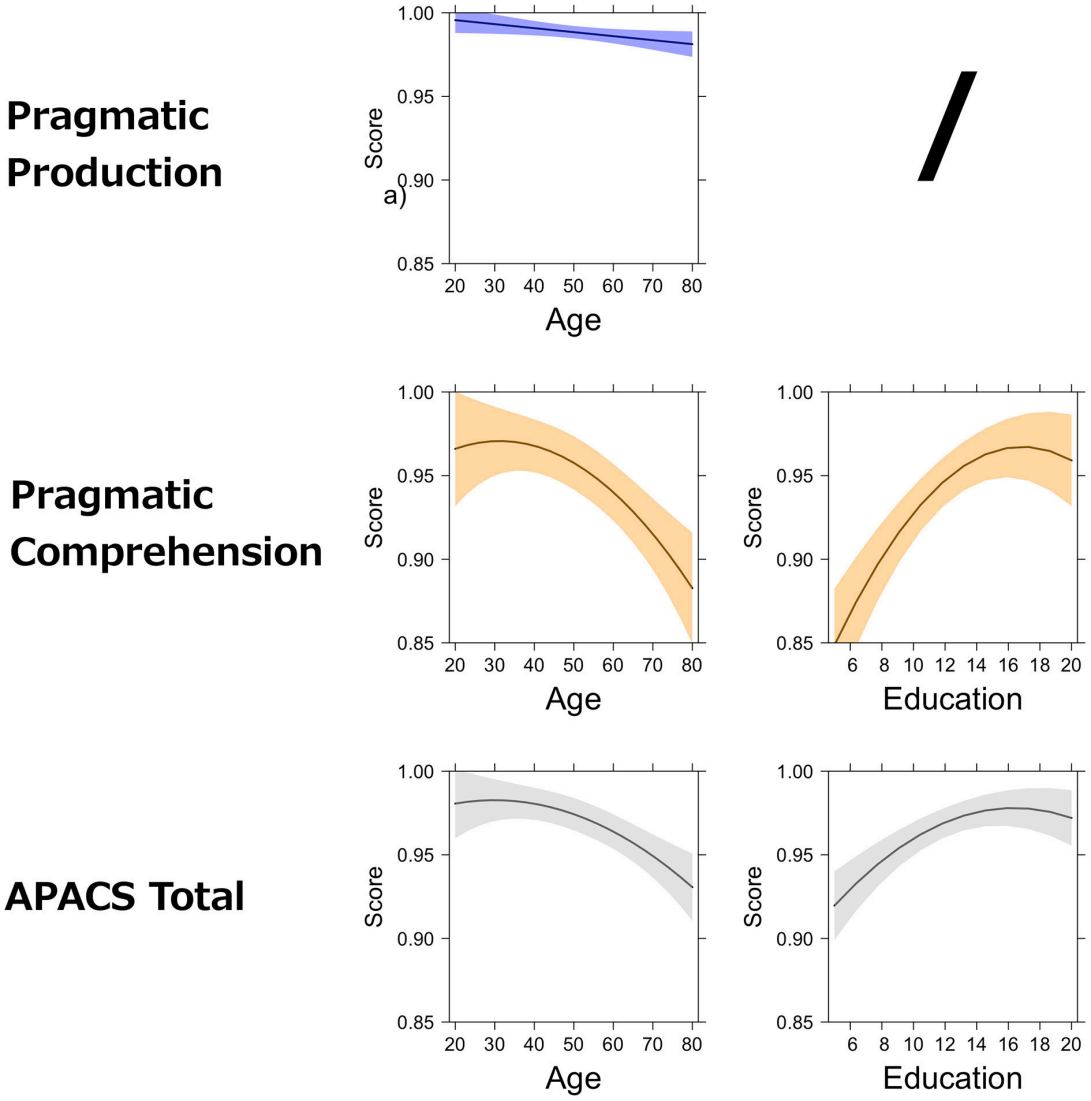
**Effect of demographic variables on APACS composite scores**. The figure shows the partial effects of age and education on APACS composite scores, as estimated by regression analysis. The figure is an array displaying the APACS composite scores (first column) and the effect of age (second column) and education (third column). A slash (“/”) indicates that the effect was not significant in the regression analysis. The black line in each plot represents the predicted score at the APACS composite score. The colored bands around the line represent point-wise confidence bands around the prediction. Light blue is used for the Pragmatic Production score. Light orange is used for the Pragmatic Comprehension score. Gray is used for the APACS Total score.

The models resulting from this procedure are reported in Table 7 and graphically represented in Figure 2 (for the APACS tasks) and Figure 3 (for the APACS composite scores). Results show a consistent pattern of age and education across APACS tasks and scores, but with some differences. Age and education showed some general effects, whereas gender never was a significant predictor. In Interview, the effect indicates that as age decreases the performance slightly decreases. In Description, no variable was significant. This means that the performance on this task is consistent across all the healthy participants, regardless of age, education, and gender. In Narratives a significant linear effect and quadratic effect of education were observed. These results indicate that performance on Narratives increases as education increases, but reaching a maximum at 16 years of education and then becoming stable. Performance in Figurative Language 1 was linearly related to both age and education, with a negative effect of age and a positive effect of education. In Humor, both age and education showed a non-linear (i.e., quadratic) relation. Age effect on Humor is slightly positive from 20 to 40 years and then negative from 40 to 89 years. The education effect on Humor is positive but, similarly to Narratives, reaches a plateau and becomes stable around 16 years. For Figurative Language 2, age had a negative linear effect, while education had a positive linear effect (similarly to Figurative Language 1 task). For the Pragmatic Production composite score only a negative effect of age was found, reflecting the effect of the Interview task on the composite score. For the Pragmatic Comprehension and APACS Total scores, both quadratic effects of age and education were found. For these two scores, age had almost no influence from 19 to 40 years, but then it showed a negative effect. Education had a positive effect, reaching a maximum around 16 years.

**Table 7:** **Effect of demographic variables on APACS tasks and composite scores**.

**Task or composite score**	**Term**	**Estimate (Standard Error)**	***t*-value**	***p*-value**	**Model *R*^2^**
Interview	Intercept	44.17 (0.31)	143.86	< 0.001^*^	0.03
	Age	−0.01 (0.006)	−2.27	0.03^*^	
Description	No significant variables				
Narratives	Intercept	44.63 (1.75)	25.50	< 0.001^*^	0.21
	Education	1.08 (0.28)	3.88	< 0.001^*^	
	Education^2^	−0.03 (0.01)	−2.96	0.004^*^	
Figurative Language 1	Intercept	14.53 (0.45)	32.00	< 0.001^*^	0.19
	Age	−0.02 (0.005)	−3.00	0.003^*^	
	Education	0.06 (0.02)	2.83	0.005^*^	
Humor	Intercept	3.11 (0.94)	3.30	0.001^*^	0.30
	Age	0.05 (0.03)	1.94	0.05	
	Age^2^	−0.0007 (0.0003)	−2.68	0.008^*^	
	Education	0.35 (0.10)	3.47	< 0.001^*^	
	Education^2^	−0.01 (0.004)	−2.85	0.005^*^	
Figurative Language 2	Intercept	26.95 (1.44)	18.72	< 0.001^*^	0.24
	Age	−0.05 (0.02)	−3.22	0.002^*^	
	Education	0.22 (0.06)	3.48	< 0.001^*^	
Pragmatic Production	Intercept	1.00 (0.006)	169.32	< 0.001^*^	0.03
	Age	−0.0002 (0.0001)	−2.14	0.03^*^	
Pragmatic Comprehension	Intercept	0.7 (0.06)	11.98	< 0.001^*^	0.42
	Age	0.003 (0.002)	1.35	0.18	
	Age^2^	−0.00004 (0.00002)	−2.26	0.03^*^	
	Education	0.03 (0.006)	4.55	< 0.001^*^	
	Education^2^	−0.0008 (0.0002)	−3.61	< 0.001^*^	
APACS Total	Intercept	0.85 (0.04)	23.75	< 0.001^*^	0.36
	Age	0.001 (0.001)	1.20	0.23	
	Age^2^	−0.00002 (0.00001)	−2.09	0.04^*^	
	Education	0.01 (0.004)	3.92	< 0.001^*^	
	Education^2^	0.0005 (0.0001)	−3.20	0.002^*^	

The following information is provided in the table: task or score name (first column); name of the term in the regression model (second column); coefficient estimate and standard error within round brackets (third column); t-value associated with the term (fourth column); p-value with stars “

* *” denoting significant terms (fifth column); adjusted R^2^ (sixth column)*.

### Cut-offs

Cut-offs were calculated for each APACS task and for the three composite scores. Rather than stratifying arbitrarily for age, education, and gender, we used a regression approach to build demographic correct norms, by means of the method proposed by Crawford and Garthwaite (2006). This method relies on the same mathematical formulas already used to identify thresholds for significant changes. Here the score of a participant is predicted from the demographic variables (i.e., age and education) of that participant, using the regression models reported in Table 7. A crucial issue when using regression-based norms is the problem of the estimate for extreme values of the predictors (in this case age and education) that could be biased as a consequence of regression model estimates. An important feature of the method by Crawford and Garthwaite is that it takes into account this problem and is also specifically designed to compare a single case with a control group^4^. Cut-offs are reported in the Supplementary Tables 3 in Supplemental Data Sheet 2: APACS-Data Tables and Cut-offs.

## Discussion

This study presents the psychometric properties and normative data of the APACS test, a new tool to evaluate pragmatic competence taking into account discourse and non-literal language through a set of 6 tasks.

APACS shows a satisfactory reliability, with acceptable internal consistency for all tasks (all Cronbach's alphas ≥ 0.60) and good test-retest reliability for almost all tasks and composite scores. A low test-retest reliability was found only for the Narratives task (*r* = 0.19), probably due to a combination of ceiling and practice effect in the test-retest sample. A factor analysis on APACS scores showed a meaningful pattern of results, with two factors accounting for task variance. One factor presumably reflects the ability to interpret figurative meanings such as idioms, metaphors, and proverbs, whereas the other factor seems related especially to pragmatic processes in detecting humor. The results of the factor analysis bring support to the construct validity of APACS, as composed by tasks tapping on different facets of the pragmatic competence. We further inspected the validity of APACS by focusing on the content validity as rated by five judges. Overall, the judges gave excellent rates to APACS items and scores, supporting the content validity of the test. When compared to other tests for pragmatic abilities, APACS has analogous values of internal consistency and very good content validity (Sacco et al., 2008). In addition, APACS is one of the few tests for which test-retest reliability is also available, which further supports the precision of the assessment instrument.

Construct validity results are especially interesting and deserve further discussion. The factorial structure of APACS evidenced two factors, one loading especially on figurative language and the other on humor. As a first consideration, this seems to confirm the view that pragmatics is not a monolithic component, and that the different pragmatic processes involved (i.e., the inferential load) might vary across tasks. Moreover, this two-factorial structure is a good starting point for discussing the role of the underlying cognitive substrates of pragmatics. There is compelling evidence on the important role of Theory of Mind and social cognition in general in inferring the speaker's intended meaning in Humor and related phenomena (e.g., sarcasm and irony) (Vrticka et al., 2013). Other literature points to the role of executive functions (like working memory and set-shifting) in humor comprehension (Bozikas et al., 2007). Hence, the second factor might be especially linked to Theory of Mind and to a lesser degree to executive functions. Note that the second factor loads also to Narratives, which is another domain in which Theory of Mind might be of some importance, especially in monitoring the protagonists' perspective (Mason and Just, 2009). The first factor, on the other hand, might be especially linked to executive functions, e.g., inhibition of inappropriate literal interpretation (Papagno and Romero Lauro, 2010) and to a lesser degree to Theory of Mind. Indeed, one might argue that only a basic ability to represent mental states is necessary for understanding metaphors (Langdon et al., 2002). We want to emphasize that this is only one of the possible interpretations of our factors in terms of cognitive substrates and that independent empirical research is needed to support this interpretation. This independent empirical research should not only focus on a normal population, but also on pathological groups. Due to the patient's cognitive and social abilities decline, a different factorial structure might emerge when studying APACS in clinical populations. This attempt to define the cognitive substrates of pragmatics is a topic of major interest, with important theoretical consequences, since some theorists describe pragmatic interpretation as essentially an exercise in mind-reading, involving inferential attribution of intentions, and argue that pragmatics is a submodule of Theory of Mind evolved for communication (Sperber and Wilson, 2002). Conversely, others argue that pragmatics is best described as a complex domain interfacing with different cognitive systems (Stemmer, 2008). Interestingly, neuroimaging evidence showed that pragmatics and Theory of Mind share important networks of activations, specifically at the level of the temporo-parietal connections (Catani and Bambini, 2014; Hagoort and Levinson, 2014). As already said, our normative data do not offer the possibility to speculate further but definitely point to the possibility of APACS to shed light on the issue of the cognitive substrates of pragmatics.

Besides the factor analysis reported here, further corroboration for the construct validity of APACS comes from an exploratory study that compared 39 patients with schizophrenia and 32 healthy controls on the APACS test (Bosia et al., 2015). In this study, patients showed an impaired performance in all APACS tasks, falling below the 5th percentile of data from the control group. The highest effect sizes of the impairment were observed in Interview, Narratives and Figurative Language 2 tasks. These findings show that APACS is a useful tool to detect the well-known pragmatic deficit in schizophrenia.

The effect of demographic variables was investigated in APACS by means of regressions, which showed a consistent pattern across tasks. Age and education influenced almost all APACS tasks and composite scores, with a negative effect of age and a positive effect of education. These results are consistent with what is commonly observed in many neuropsychological tests (Strauss et al., 2006). Moreover, these results match with experimental research on the effects of age on specific pragmatic abilities, where aging is showed to affect the comprehension of jokes (Mak and Carpenter, 2007), written text (Borella et al., 2011) and the neural response for metaphor (Bonnaud et al., 2002; Mejía-Constaín et al., 2010). Studies on aging and pragmatics also pointed out that the decline in pragmatic performance in the aged population is probably related to a conundrum of other cognitive abilities (Mak and Carpenter, 2007), and it is possibly reduced once we factor out the working memory load (Borella et al., 2007). These results further highlight the importance of exploring the cognitive substrates of pragmatics, complementing the assessment of pragmatic abilities with neuropsychological tests targeting executive functions and social cognition. Interestingly, studies showed that the ability of comprehending figurative uses of language improves during adolescence, reaching a plateau in adulthood (Nippold et al., 1997), which remains relatively stable in elderly subjects with a high education level (Bonnaud et al., 2002). In APACS we found an interplay between age and education that could be consistent with these findings.

Finally, we reported cut-offs for clinical purposes, calculated by using state-of-the-art techniques based on regression analysis (Crawford and Howell, 1998b; Crawford and Garthwaite, 2006). Importantly, and innovatively with respect to previous tests, we also provided thresholds to detect significant changes, which allow to determine if a single patient has improved or worsened at two repeated measurements. Thresholds for significant change can be used to test if a patient changes after a treatment or after a neurosurgical intervention, or to test if the patient shows a decline in pragmatic abilities over time.

Overall, this study shows that APACS is a valuable tool to detect impairments in verbal pragmatic abilities, which could be employed for research as well as for clinical purposes. To this respect, the total duration of the test (around 35–40 min) and the use of traditional tasks and scoring systems not requiring effortful training on the clinician's side should add to the feasibility of APACS in clinical settings. In terms of clinical utility, the importance of a test assessing pragmatic abilities like APACS comes from two main considerations. First, a large body of research reports communicative breakdowns in specific pragmatic tasks across several clinical populations, from schizophrenia to traumatic brain injuries, where deficits are documented for instance in metaphor comprehension or discourse and conversation (Martin and McDonald, 2003; Brüne and Bodenstein, 2005). The number of clinical populations that exhibit pragmatic impairments has been recently expanded with data from neurodegenerative diseases, including fronto-temporal dementia and amyotrophic lateral sclerosis (Orange and Hillis, 2012; Ash et al., 2014). APACS is suitable for use in both psychiatric and neurological patients, including patients with dysarthria and other production difficulties, as it contains tasks that do not require production and separate cut-offs are provided for each task. Second, pragmatics is intimately related to communication, and it lies at the heart of our social life, with high impact on the individual's life and on society at large. A compact test like APACS could contribute to providing a complete picture of the pragmatic competence in the different clinical populations, targeting a vital domain in the patient's social life, and ultimately leading to a more precise characterization of the different clinical profiles.

An important aspect deserving consideration for future uses of APACS is related to the description of the cognitive substrates of pragmatic abilities. Factor analysis offered hints in this direction, with Figurative Language tasks and Humor clustering separately, possibly in relation to different cognitive substrates. Coupling APACS with neuropsychological tests could contribute to clarifying how cognitive functions are involved in pragmatics. Although clearly unified by their close relation to the communicative context, the pragmatic tasks included in APACS might differ from each other and might differently tax on cognitive abilities. Research on patients might shed light on the inventory of pragmatic phenomena by highlighting specific interplays of communicative performance and neurocognitive deficits.

To conclude, with APACS we aim at providing a tool that could promote the inclusion of pragmatics in the clinical assessment practice, as a relevant dimension in defining the patient's cognitive profile, as well as research on the neurocognitive underpinning of the typically human abilities of adjusting communicative behavior to context.

## Author contributions

The authors designed the APACS test and run the study together. VB is especially responsible for the pragmatic aspects of the test and GA for the statistical analyses.

### Conflict of interest statement

The authors declare that the research was conducted in the absence of any commercial or financial relationships that could be construed as a potential conflict of interest.

## Footnotes

^1^Note that, in including discourse and non-literal language understanding, APACS provides a view of pragmatic abilities which is in line with the recent classification of neurodevelopmental Semantic-Pragmatic Disorders in the DSM-5, specifically sharing information in criterion 1, story-telling and conversation in criterion 3 and non-literal meaning in criterion 4.

^2^In calculating the Cronbach's alpha of Figurative Language 1 task, we removed two items almost at ceiling, i.e., Item 1 and Item 2. Nevertheless, we decided to keep these items in the final version of APACS, because they are not associated to a ceiling performance in patients and thus might be useful to detect impairment (Bosia et al., 2015). Alpha including the two items was 0.54.

^3^Notably, in a joint analysis on unpublished data that included both patients with schizophrenia and healthy controls tested with APACS, the Narratives task shows a satisfying value of Test-Retest reliability of 0.76.

^4^For the Description task, since no predictor was significant in the regression analysis, we used the formula by Crawford and Howell (1998b). This formula allows to calculate cut-offs analogous to those obtained with the regression method by Crawford and Garthwaite (2006).

## References

[B1] Amanzio M. Geminiani G. Leotta D. Cappa S. (2008). Metaphor comprehension in Alzheimer's disease: novelty matters. Brain Lang. 107, 1–10. 10.1016/j.bandl.2007.08.003 17897706

[B2] Angeleri R. Bosco F. M. Gabbatore I. Bara B. G. Sacco K. (2012). Assessment battery for communication (ABaCo): normative data. Behav. Res. Methods 44, 845–861. 10.3758/s13428-011-0174-9 22180102

[B3] Ash S. Menaged A. Olm C. McMillan C. T. Boller A. Irwin D. J.. (2014). Narrative discourse deficits in amyotrophic lateral sclerosis. Neurology 83, 520–528. 10.1212/WNL.0000000000000670 24991038 PMC4142005

[B4] Bambini V. (2010). Neuropragmatics: a foreword. Ital. J. Linguist. 22, 1–20.

[B5] Bambini V. Bara B. G. (2012). Neuropragmatics, in Handbook of Pragmatics, eds Östman J.-O. Verschueren J. (Amsterdam: John Benjamins), 1–21.

[B6] Bambini V. Ghio M. Moro A. Schumacher P. B. (2013). Differentiating among pragmatic uses of words through timed sensicality judgments. Front. Psychol. 4:938. 10.3389/fpsyg.2013.00938 24391608 PMC3867823

[B7] Bonnaud V. Gil R. Ingrand P. (2002). Metaphorical and non-metaphorical links: a behavioral and ERP study in young and elderly adults. Neurophysiol. Clin. 32, 258–268. 10.1016/S0987-7053(02)00307-6 12448183

[B8] Borella E. De Beni R. De Ribaupierre A. (2007). La comprensione del testo in giovani e anziani: un'abilità stabile? G. Ital. Psicol. 2, 407–426. 10.1421/24629

[B9] Borella E. Ghisletta P. de Ribaupierre A. (2011). Age differences in text processing: the role of working memory, inhibition, and processing speed. J. Gerontol. B. Psychol. Sci. Soc. Sci. 66, 311–320. 10.1093/geronb/gbr002 21339301

[B10] Borovsky A. Saygin A. P. Bates E. Dronkers N. (2007). Lesion correlates of conversational speech production deficits. Neuropsychologia 45, 2525–2533. 10.1016/j.neuropsychologia.2007.03.023 17499317 PMC5610916

[B11] Bosco F. M. Angeleri R. Zuffranieri M. Bara B. G. Sacco K. (2012). Assessment Battery for Communication: development of two equivalent forms. J. Commun. Disord. 45, 290–303. 10.1016/j.jcomdis.2012.03.002 22483360

[B12] Bosia M. Arcara G. Moro A. Cavallaro R. Bambini V. (2015). Pragmatic abilities across symptoms dimensions in schizophrenia, in Studi Italiani di Linguistica Teorica e Applicata, XLIV.

[B13] Bozikas V. P. Kosmidis M. H. Giannakou M. Anezoulaki D. Petrikis P. Fokas K.. (2007). Humor appreciation deficit in schizophrenia: the relevance of basic neurocognitive functioning. J. Nerv. Ment. Dis. 195, 325–331. 10.1097/01.nmd.0000243798.10242.e2 17435483

[B14] Brownell H. H. Michel D. Powelson J. Gardner H. (1983). Surprise but not coherence: sensitivity to verbal humor in right-hemisphere patients. Brain Lang. 18, 20–27. 10.1016/0093-934X(83)90002-0 6839130

[B15] Brüne M. Bodenstein L. (2005). Proverb comprehension reconsidered - “Theory of mind” and the pragmatic use of language in schizophrenia. Schizophr. Res. 75, 233–239. 10.1016/j.schres.2004.11.006 15885515

[B16] Bryan K. L. (1995). The Right Hemisphere Language Battery, 2nd Edn. London: Whurr Publisher.

[B17] Cardillo E. R. Schmidt G. L. Kranjec A. Chatterjee A. (2010). Stimulus design is an obstacle course: 560 matched literal and metaphorical sentences for testing neural hypotheses about metaphor. Behav. Res. Methods 42, 651–664. 10.3758/BRM.42.3.651 20805587 PMC2952404

[B18] Catani M. Bambini V. (2014). A model for social communication and language evolution and development (SCALED). Curr. Opin. Neurobiol. 28, 165–171. 10.1016/j.conb.2014.07.018 25156623

[B19] Champagne-Lavau M. Stip E. Joanette Y. (2007). Language functions in right-hemisphere damage and schizophrenia: apparently similar pragmatic deficits may hide profound differences. Brain 130, e67. 10.1093/brain/awl311 17235120

[B20] Clark C. N. Nicholas J. M. Henley S. M. D. Downey L. E. Woollacott I. O. Golden H. L.. (2015). Humour processing in frontotemporal lobar degeneration: a behavioural and neuroanatomical analysis. Cortex 69, 47–59. 10.1016/j.cortex.2015.03.024 25973788 PMC4534772

[B21] Collie A. Darby D. G. Falleti M. G. Silbert B. S. Maruff P. (2002). Determining the extent of cognitive change after coronary surgery: a review of statistical procedures. Ann. Thorac. Surg. 73, 2005–2011. 10.1016/S0003-4975(01)03375-6 12078822

[B22] Crawford J. R. Garthwaite P. H. (2006). Comparing patients' predicted test scores from a regression equation with their obtained scores: a significance test and point estimate of abnormality with accompanying confidence limits. Neuropsychology 20, 259–271. 10.1037/0894-4105.20.3.259 16719619

[B23] Crawford J. R. Howell D. C. (1998a). Regression equations in clinical neuropsychology: An evaluation of statistical methods for comparing predicted and obtained scores. J. Clin. Exp. Neuropsych. 20, 755–762. 10.1076/jcen.20.5.755.1132 10079050

[B24] Crawford J. R. Howell D. C. (1998b). Comparing an individual's test score against norms derived from small samples. Clin. Neuropsychol. 12, 482–486. 10.1076/clin.12.4.482.7241

[B25] Crockford C. Lesser R. (1994). Assessing functional communication in aphasia: clinical utility and time demands of three methods. Eur. J. Disord. Commun. 29, 165–182. 10.3109/13682829409041490 7532476

[B26] Ferstl E. C. (2010). Neuroimaging of text comprehension: where are we now? Ital. J. Linguist. 1, 61–88.

[B27] Ferstl E. C. Walther K. Guthke T. von Cramon D. Y. (2005). Assessment of story comprehension deficits after brain damage. J. Clin. Exp. Neuropsychol. 27, 367–384. 10.1080/13803390490515784 15969358

[B28] Gardner H. Brownell H. H. (1986). Right Hemisphere Communication Battery. Boston, MA: Psychology Service.

[B29] Grice H. P. (1975). Logic and conversation, in Syntax and Semantics, Vol. III: Speech Acts, eds Cole P. Morgan J. L. (New York, NY: Academic Press), 41–58.

[B30] Guazzotti P. Oddera M. F. (2006). Il Grande Dizionario dei Proverbi Italiani. Bologna: Zanichelli.

[B31] Hagoort P. Levinson S. C. (2014). Neuropragmatics, in The Cognitive Neurosciences, ed Gazzaniga M. S. (Cambridge, MA: MIT Press), 667–674.

[B32] Jacobson N. S. Truax P. (1991). Clinical significance: a statistical approach to defining meaningful change in psychotherapy research. J. Consult. Clin. Psych. 59, 12–19. 10.1037/0022-006X.59.1.12 2002127

[B33] Joanette Y. Brownell H. H. (eds.). (1990). Discourse Ability and Brain Damage - Theoretical and Empirical. (New York, NY: Springer-Verlag).

[B34] Joanette Y. Goulet P. Hannequin D. Boeglin J. (1990). Right Hemisphere and Verbal Communication. New York, NY: Springer-Verlag.

[B35] Langdon R. Davies M. Coltheart M. A. X. (2002). Understanding minds and understanding communicated meanings in Schizophrenia. Mind Lang. 17, 68–104. 10.1111/1468-0017.00190

[B36] Levinson S. C. (1983). Pragmatics. Cambridge: Cambridge University Press.

[B37] Long A. F. Hesketh A. Paszek G. Booth M. Bowen A. (2008). Development of a reliable self-report outcome measure for pragmatic trials of communication therapy following stroke: the Communication Outcome after Stroke (COAST) scale. Clin. Rehabil. 22, 1083–1094. 10.1177/0269215508090091 19052247

[B38] Lucisano P. Piemontese M. E. (1988). GULPEASE: una formula per la predizione della difficoltà dei testi in lingua italiana. Sc. Città XXXIX, 110–124.

[B39] Mak W. Carpenter B. D. (2007). Humor comprehension in older adults. J. Int. Neuropsychol. Soc. 13, 606–614. 10.1017/s1355617707070750 17521496

[B40] Marini A. Andreetta S. del Tin S. Carlomagno S. (2011). A multi-level approach to the analysis of narrative language in aphasia. Aphasiology 25, 1372–1392. 10.1080/02687038.2011.584690

[B41] Marini A. Zettin M. Galetto V. (2014). Cognitive correlates of narrative impairment in moderate traumatic brain injury. Neuropsychologia 64C, 282–288. 10.1016/j.neuropsychologia.2014.09.042 25281884

[B42] Martin I. McDonald S. (2003). Weak coherence, no theory of mind, or executive dysfunction? Solving the puzzle of pragmatic language disorders. Brain Lang. 85, 451–466. 10.1016/S0093-934X(03)00070-1 12744957

[B43] Mason R. A. Just M. A. (2009). The role of the theory-of-mind cortical network in the comprehension of narratives. Lang. Linguist. Compass 3, 157–174. 10.1111/j.1749-818X.2008.00122.x 19809575 PMC2756681

[B44] McDonald S. (2008). Frontal lobes and language, in Handbook of the Neuroscience of Language, eds Stemmer B. Whitaker H. (New York, NY: Elsevier), 289–297.

[B45] Mejía-Constaín B. Monchi O. Walter N. Arsenault M. Senhadji N. Joanette Y. (2010). When metaphors go literally beyond their territories: the impact of age on figurative language. Ital. J. Linguist. 22, 41–60.

[B46] Nippold M. A. Uhden L. D. Schwarz I. E. (1997). Proverb explanation through the lifespan: a developmental study of adolescents and adults. J. Speech. Lang. Hear. Res. 40, 245–253. 10.1044/jslhr.4002.245 9130197

[B47] Orange J. B. Hillis A. E. (2012). Language profiles in amyotrophic lateral sclerosis, in Amyotrophic Lateral Sclerosis and the Frontotemporal Dementias (Oxford: Oxford University Press), 78–92.

[B48] Papagno C. Cappa S. F. Forelli A. Garavaglia G. Capitani E. (1995). La comprensione non letterale del linguaggio: taratura di un test di comprensione di metafore e di espressioni idiomatiche. Arch. Psicol. Neurol. Psichiatr. 4, 402–420.

[B49] Papagno C. Romero Lauro L. J. (2010). The neural basis of idiom processing: neuropsychological, neurophysiological and neuroimaging evidence. Ital. J. Linguist. 22, 21–40.

[B50] Penn C. (1985). The profile of communicative appropriateness: a clinical tool for the assessment of pragmatics. S. Afr. J. Commun. Disord. 32, 18–23. 3832443

[B51] Premack D. Woodruff G. (1978). Does the chimpanzee have a theory of mind? Behav. Brain Sci. 1, 515–526. 10.1017/S0140525X00076512

[B52] Prutting C. A. Kirchner D. M. (1987). A clinical appraisal of the pragmatic aspects of language. J. Speech Hear. Disord. 52, 105–119. 10.1044/jshd.5202.105 3573742

[B53] R Core Team (2015). R: A Language and Environment for Statistical Computing. Vienna: R Foundation for Statistical Computing Available online at: http://www.R-project.org/

[B54] Rinaldi M. C. Marangolo P. Lauriola M. (2004). BLED SantaLucia. Batteria sul Linguaggio dell'Emisfero Destro SantaLucia. Firenze: Giunti O.S.

[B55] Sacco K. Angeleri R. Bosco F. M. Colle L. Mate D. Bara B. G. (2008). Assessment battery for communication—ABaCo: a new instrument for the evaluation of pragmatic abilities. J. Cogn. Sci. (Seoul). 9, 111–157. 10.17791/jcs.2008.9.2.111

[B56] Semenza C. (2002). Lexical-semantic disorders in aphasia, in Handbook of Clinical and Experimental Neuropsychology, eds Denes G. Pizzamiglio L. (Hove: Psychology Press), 215–244.

[B57] Sperber D. Wilson D. (2002). Pragmatics, modularity and mind-reading. Mind Lang. 17, 3–23. 10.1111/1468-0017.00186

[B58] Sperber D. Wilson D. (2005). Pragmatics, in Oxford Handbook of Contemporary Philosophy, eds Jackson F. Smith M. (Oxford: Oxford University Press), 468–501.

[B59] Spinnler H. Tognoni G. (1987). Standardizzazione e taratura italiana di test neuropsicologici. Ital. J. Neurol. Sci. 8, 1–120. 3330072

[B60] Stemmer B. (2008). Neuropragmatics: disorders and neural systems, in Handbook of the Neuroscience of Language, eds Stemmer B. Whitaker H. A. (New York, NY: Elsevier), 175–187.

[B61] Strauss E. Sherman E. M. Spreen O. (2006). A Compendium of Neuropsychological Tests: Administration, Norms, and Commentary. Oxford: Oxford University Press.

[B62] Tabossi P. Arduino L. Fanari R. (2011). Descriptive norms for 245 Italian idiomatic expressions. Behav. Res. Methods 43, 110–123. 10.3758/s13428-010-0018-z 21287129

[B63] Tavano A. Côté H. Ferré P. Ska B. Joanette Y. (2013). Protocollo MEC - Protocollo Montréal per la Valutazione delle Abilità Comunicative. Milan: Springer.

[B64] Vrticka P. Black J. M. Reiss A. L. (2013). The neural basis of humour processing. Nat. Rev. Neurosci. 14, 860–868. 10.1038/nrn3566 24169937

[B65] Winner E. Gardner H. (1977). The comprehension of metaphor in brain-damaged patients. Brain 100, 717–729. 10.1093/brain/100.4.717 608117

[B66] Yus F. (2008). A relevance-theoretic classification of jokes. Lodz Pap. Pragmat. 4, 131–157. 10.2478/v10016-008-0004-4

