# Supplementary material for: A Test for the Assessment of Pragmatic Abilities and Cognitive Substrates (APACS): Normative Data and Psychometric Properties
Source: Front Psychol. 2016 Feb 12;7:70. doi: 10.3389/fpsyg.2016.00070 (PMC4751735; doi:10.3389/fpsyg.2016.00070)
Supplement: Supplementary file 1 [file SupplementalDataSheet1APACS-ItemExamples.PDF]

Supplementary Material

Supplemental Data Sheet 1: APACS-Item Examples

A test for the Assessment of Pragmatic Abilities and Cognitive Substrates (APACS): Normative data and psychometric properties

Giorgio Arcara and Valentina Bambini*

*** Correspondence:** Valentina Bambini: valentina.bambini@iusspavia.it

Below we provide one item from each APACS task (except for the Interview task, which is based on the patient's speech production), from the original Italian version, and with literal English translation.

Description – Item 9

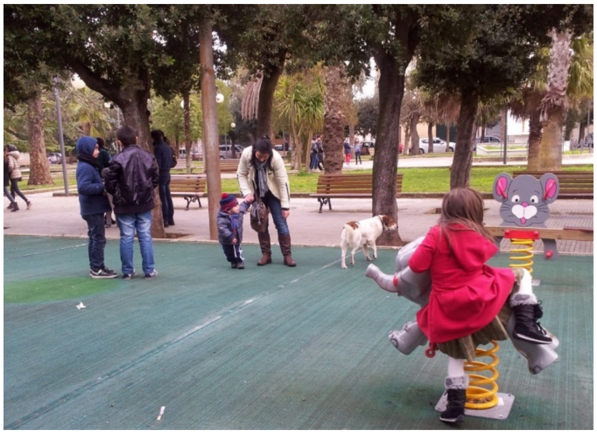	Italian<br><br>Elementi attesi:<br>• Giardini<br>• Bambini<br>• Giocare
	English<br><br>Expected elements:<br>• Playground<br>• Children<br>• Playing

Narratives – Item 2

Italian	English
Il comitato dei pendolari lombardi ha condotto un'indagine sulle corse dei treni regionali dalle 6 alle 9 del mattino. Nella giornata di ieri, su 52 treni, ben 49 hanno avuto un ritardo tra i 5 e i 30 minuti. Ogni mattina lo scenario si ripete e i viaggiatori hanno un diavolo per capello. Intervistato, il portavoce Mario Ginestri ha detto che i pendolari avvieranno una protesta formale contro i treni lumaca. Intanto, la compagnia dei trasporti ha promesso interventi per la manutenzione delle linee, attesi per la prossima estate.<br><br>• Domanda globale<br>*Di cosa si parla in questa notizia?*<br>• Domanda specifica 1<br>*I pendolari sono soddisfatti dei treni?*<br>• Domanda specifica 2<br>*Gli interventi di manutenzione sono previsti per l'inverno?*<br>• Domanda su significato figurato 1<br>*Che cosa significa che i viaggiatori hanno un diavolo per capello?*<br>• Domanda su significato figurato 2<br>*Che cosa significa che i pendolari protestano contro i treni lumaca? Che cosa sono i treni lumaca?*	*The Lombardy commuters committee conducted a survey on the regional train service between 6 and 9 in the morning. Yesterday, out of a total of 52 trains, as many as 49 suffered delays ranging from 5 to 30 minutes. The same scenario is repeated every morning and travellers are beside themselves with rage. In an interview, the spokesman Mario Ginestri said that commuters will initiate a formal protest against the snail trains. Meanwhile, the transport company has promised measures for the maintenance of the tracks, expected for next summer.*<br><br>• Global question<br>*What is the topic of this story?*<br>• Specific question 1<br>*Are commuters satisfied with the train service?*<br>• Specific question 2<br>*Are measures for maintenance planned for next winter?*<br>• Question of figurative language 1<br>*What does it mean when we say that travellers have blown a gasket?*<br>• Question on figurative language 2<br>*What does it mean when we say that commuters protest against the snail trains? What are snail trains?*

Figurative Language 1 – Item 6 (metaphor)

Italian	English
Ho appena visto una corsa di formula uno. Certe automobili sono frecce.<br><br>Opzioni:<br>• Interpretazione figurata (corretta)<br>*Certe automobili sono veloci*<br>• Interpretazione non relata (scorretta)<br>*Certe automobili sono lussuose*<br>• Interpretazione letterale (scorretta)<br>*Certe automobili sono appuntite*	*I have just seen a F1 match. Some cars are arrows.*<br><br>Options:<br>• Figurative interpretation (correct)<br>*Some cars are fast*<br>• Unrelated interpretation (incorrect)<br>*Some cars are luxurious*<br>• Literal interpretation (incorrect)<br>*Some cars are pointy*

Humor – Item 6

Italian	English
La signora Rossi visita la casa di un'amica. Di fronte ad un bellissimo mobile antico, esclama: "Che magnifico mobile! Di che epoca è?" E l'amica risponde:<br>Opzioni:<br>• Finale umoristico (corretto)<br>*Dell'epoca in cui avevamo i soldi*<br>• Finale coerente ma non umoristico (scorretto)<br>*Del settecento inglese*<br>• Finale non relato (scorretto)<br>*Le tarme del legno sono un bel problema*	*Mrs Rossi calls on a friend of hers. One seeing a beautiful piece of antique furniture, she exclaims: "What a splendid piece! When does it date back to?" And her friend replies:*<br>Options:<br>• Funny ending (correct)<br>*To when we used to have money*<br>• Straightforward ending (incorrect)<br>*To the eighteenth century*<br>• Unrelated ending (incorrect)<br>*Woodworm is such a problem*

Figurative Language 2 – Item 15 (proverb)

Italian	English
Una rondine non fa primavera	*One swallow does not make a summer*
