# Supplementary material for: A Test for the Assessment of Pragmatic Abilities and Cognitive Substrates (APACS): Normative Data and Psychometric Properties
Source: Front Psychol. 2016 Feb 12;7:70. doi: 10.3389/fpsyg.2016.00070 (PMC4751735; doi:10.3389/fpsyg.2016.00070)
Supplement: Supplementary file 2 [file SupplementalDataSheet2APACS-DataTablesandCut-offs.PDF]

Supplementary Material

Supplemental Data Sheet 2: APACS-Data Tables and Cut-offs

A test for the Assessment of Pragmatic Abilities and Cognitive Substrates (APACS): Normative data and psychometric properties

Giorgio Arcara and Valentina Bambini*

*** Correspondence:** Valentina Bambini: valentina.bambini@iusspavia.it

Supplementary Tables 1. Descriptive Statistics of APACS results

The tables report the means, standard deviations, median, minimum, maximum, kurtosis, skewness, first quartile, and third quartile for APACS scores in the normative data group of 119 participants, divided in four age and education groups.

Supplementary Table 1.1. Descriptive statistics for the first age and education group

N = 35<br>Age < 55; Education ≤ 13									
**Task or composite score**	**Mean**	**SD**	**Median**	**Min**	**Max**	**Kurtosis**	**Skewness**	**Q1**	**Q3**
Interview	43.46	1.20	44	39	44	5.10	-2.40	43.5	44
Description	47.57	1.27	48	43	48	5.53	-2.64	48	48
Narratives	53.40	1.90	54	46	56	4.52	-1.87	53	55
Figurative Language 1	14.77	0.55	15	13	15	3.76	-2.20	15	15
Humor	6.51	0.70	7	5	7	-0.30	-1.04	6	7
Figurative Language 2	27.69	3.32	28	16	30	4.04	-2.10	27.5	30
Pragmatic Production	0.99	0.02	1	0.94	1	0.56	-1.52	0.99	1
Pragmatic Comprehension	0.95	0.05	0.96	0.77	1	4.51	-2.05	0.93	0.98
APACS Total	0.97	0.03	0.97	0.86	1	4.42	-2.09	0.96	0.99

Supplementary Table 1.2. Descriptive statistics for the second age and education group

N = 30
Age < 55; Education > 13

Task or composite score	Mean	SD	Median	Min	Max	Kurtosis	Skewness	Q1	Q3
Interview	43.8	0.55	44	42	44	4.91	-2.49	44	44
Description	47.4	1.4	48	44	48	1.64	-1.86	48	48
Narratives	54.1	2.22	55	48	56	1.43	-1.46	53.25	56
Figurative Language 1	14.9	0.31	15	14	15	4.58	-2.53	15	15
Humor	6.83	0.46	7	5	7	6.41	-2.64	7	7
Figurative Language 2	28.6	1.94	30	23	30	0.49	-1.23	28	30
Pragmatic Production	0.99	0.02	1	0.95	1	0.93	-1.55	0.99	1
Pragmatic Comprehension	0.97	0.03	0.98	0.92	1	-0.95	-0.67	0.96	0.99
APACS Total	0.98	0.02	0.99	0.95	1	-0.8	-0.8	0.97	1

Supplementary Table 1.3. Descriptive statistics for the third age and education group

N = 40
Age ≥ 55; Education ≤ 13

Task or composite score	Mean	SD	Median	Min	Max	Kurtosis	Skewness	Q1	Q3
Interview	43.42	1.17	44	39	44	4.51	-2.23	43	44
Description	47.5	1.72	48	38	48	20.48	-4.4	48	48
Narratives	51.6	3.97	52.5	37	56	2.48	-1.4	49	55
Figurative Language 1	13.95	1.34	14	9	15	3.16	-1.72	13.75	15
Humor	5.85	1.48	6	0	7	4.14	-1.84	5	7
Figurative Language 2	25.52	3.4	26	16	30	0.4	-0.95	24	28
Pragmatic Production	0.99	0.02	1	0.9	1	6.46	-2.42	0.99	1
Pragmatic Comprehension	0.88	0.1	0.91	0.52	1	2.97	-1.66	0.87	0.95
APACS Total	0.94	0.06	0.95	0.71	1	5.31	-2.03	0.92	0.97

Supplementary Table 1.4. Descriptive statistics for the fourth age and education group

N = 14
Age ≥ 55; Education > 13

Task or composite score	Mean	SD	Median	Min	Max	Kurtosis	Skewness	Q1	Q3
Interview	43.21	1.31	44	40	44	0.29	-1.31	43	44
Description	46.93	1.86	48	42	48	1.05	-1.50	46.25	48
Narratives	53.14	2.85	54	45	56	1.98	-1.52	52.25	55
Figurative Language 1	14.43	0.85	15	13	15	-1.16	-0.84	14	15
Humor	6.14	1.35	6.50	2	7	3.44	-1.98	6	7
Figurative Language 2	27.36	2.47	28	22	30	-0.40	-0.84	26.25	29
Pragmatic Production	0.98	0.03	0.98	0.9	1	1.83	-1.54	0.98	1
Pragmatic Comprehension	0.93	0.07	0.95	0.72	1	1.63	-1.48	0.9	0.97
APACS Total	0.95	0.05	0.96	0.81	1	2.43	-1.7	0.95	0.98

Supplementary Tables 2. Thresholds for determining significant changes

The following tables allow to determine if a significant change occurred in APACS scores. A separate table is provided for each APACS task and composite score. The utilization of these tables assumes that two measurements from an individual (1st measurement and 2nd measurement) are available. To determine if a significant change occurred, first find the observed value at 1st measurement in the central row of the table (values in bold typeface). Then, check the column corresponding to this value in the table. The upper row reports the threshold for a significant improvement. The lower row reports the threshold for a significant worsening. If the observed score at second measurement *falls outside* these thresholds, then a significant change occurred. If the observed value falls within these thresholds (threshold values included), no change occurred. Specifically, it is estimated that at second measurement less than 5% of the population will exceed the upper thresholds and less than 5% of the population will fall below the lower thresholds.
The thresholds for significant change were determined by means of the method by Crawford & Garthwaite (2006). The range of values reported for each test is the range observed in the overall normative sample (119 participants). Gray cells indicate values extrapolated from the regressions used to define significant changes. Gray cell values are not actually observed in the smaller sample used to build the tables (19 participants) and they should be used with caution. In some cases the thresholds could not be calculated (for example if the predicted value exceeded the range of possible score values). In such cases a hyphen is reported.
If the tables cannot be used to test a specific change (for example if the observed score of a patient is not reported in the table), it is possible to build a new table by collecting data on a small control sample and applying the method (and accompanying software) by Crawford and Garthwaite (2006).

Note 1) In some cases the lower threshold at second measurement can be higher than the observed value at first measurement. This can happen if a practice effect is expected.

Note 2) The Test-Retest interval used to build these tables is 2 weeks. When used for Test-Retest intervals longer than two weeks, these tables might be over-corrected for practice effect.

Supplementary Table 2.1. Thresholds for significant changes for the Interview task.

Interview						
Upper Threshold (2nd meas.)	42	42	43	43	44	-
**Observed value (1st meas.)**	**39**	**40**	**41**	**42**	**43**	**44**
Lower Threshold (2nd meas.)	39	40	41	42	43	43

Supplementary Table 2.2. Thresholds for significant changes for the Description task.

Description											
Upper Threshold (2nd meas.)	39	40	41	42	43	44	45	47	-	-	-
**Observed value (1st meas.)**	**38**	**39**	**40**	**41**	**42**	**43**	**44**	**45**	**46**	**47**	**48**
Lower Threshold (2nd meas.)	34	35	36	38	39	40	41	43	44	45	46

Supplementary Table 2.3. Thresholds for significant changes for the Narratives task. No Upper threshold is available as a consequence of the test-retest reliability calculated from the sample (n=19).

Narratives											
Upper Threshold (2nd meas.)	-	-	-	-	-	-	-	-	-	-	-
**Observed value (1st meas.)**	**37**	**39**	**41**	**43**	**45**	**47**	**49**	**51**	**53**	**55**	**56**
Lower Threshold (2nd meas.)	48	49	49	50	51	52	52	53	53	53	53

Supplementary Table 2.4. Thresholds for significant changes for the Figurative Language 1 task.

Figurative Language 1							
Upper Threshold (2nd meas.)	12	12	13	14	14	-	-
**Observed value (1st meas.)**	**9**	**10**	**11**	**12**	**13**	**14**	**15**
Lower Threshold (2nd meas.)	11	12	12	13	14	14	15

Supplementary Table 2.5. Thresholds for significant changes for the Humor task.

Humor								
Upper Threshold (2nd meas.)	4	5	5	5	6	6	-	-
**Observed value (1st meas.)**	**0**	**1**	**2**	**3**	**4**	**5**	**6**	**7**
Lower Threshold (2nd meas.)	-	1	2	3	4	5	5	6

Supplementary Table 2.6. Thresholds for significant changes for the Figurative Language 2 task.

Figurative Language 2															
Upper Threshold (2nd meas.)	18	19	20	21	22	23	23	24	25	26	27	28	29	-	-
**Observed value (1st meas.)**	**16**	**17**	**18**	**19**	**20**	**21**	**22**	**23**	**24**	**25**	**26**	**27**	**28**	**29**	**30**
Lower Threshold (2nd meas.)	10	12	13	14	16	17	18	20	21	22	24	25	26	27	28

Supplementary Table 2.7. Thresholds for significant changes for the Pragmatic Production score.

Pragmatic Production											
Upper Threshold (2nd meas.)	0.92	0.93	0.94	0.95	0.96	0.97	0.98	0.99	-	-	-
**Observed value (1st meas.)**	**0.9**	**0.91**	**0.92**	**0.93**	**0.94**	**0.95**	**0.96**	**0.97**	**0.98**	**0.99**	**1**
Lower Threshold (2nd meas.)	0.87	0.88	0.89	0.9	0.91	0.93	0.94	0.95	0.96	0.97	0.98

Supplementary Table 2.8. Thresholds for significant changes for the Pragmatic Comprehension score.

Pragmatic Comprehension											
Upper Threshold (2nd meas.)	0.74	0.76	0.79	0.82	0.84	0.87	0.9	0.93	0.96	0.99	-
**Observed value (1st meas.)**	0.5	0.55	0.6	0.65	0.7	0.75	0.8	0.85	0.9	0.95	1
Lower Threshold (2nd meas.)	0.53	0.58	0.63	0.67	0.72	0.76	0.81	0.85	0.89	0.93	0.96

Supplementary Table 2.9. Thresholds for significant changes for the APACS Total score.

APACS Total							
Upper Threshold (2nd meas.)	0.82	0.85	0.88	0.91	0.94	0.97	-
**Observed value (1st meas.)**	0.7	0.75	0.8	0.85	0.9	0.95	1
Lower Threshold (2nd meas.)	0.73	0.77	0.82	0.86	0.9	0.94	0.98

Supplementary Tables 3. APACS cut-offs

The following tables report cut-offs for all APACS scores. A separate table is provided for each task and APACS composite score. The cut-offs were calculated with the method by Crawford and Garthwaite (2006), using regression analyses taking into account age, education and gender[1]. Since no effect of gender was found, tables are not split for gender. The tables report the cut-offs for different age and education combinations. An observed value *below* the cut-off indicates an impaired performance. Specifically, it is estimated that less than 5% of the population will obtain a score below the cut-offs reported in the tables.

Supplementary Table 3.1. Cut-offs for the Interview Task.

Interview – cut-offs

	Age												
**Education**	20	25	30	35	40	45	50	55	60	65	70	75	80
5	42	42	42	42	42	42	42	42	42	42	41	41	41
8	42	42	42	42	42	42	42	42	42	42	41	41	41
13	42	42	42	42	42	42	42	42	42	42	41	41	41
18	42	42	42	42	42	42	42	42	42	42	41	41	41
23	42	42	42	42	42	42	42	42	42	42	41	41	41

Supplementary Table 3.2. Cut-offs for the Description Task.

Description – cut-offs

	Age												
**Education**	20	25	30	35	40	45	50	55	60	65	70	75	80
5	45	45	45	45	45	45	45	45	45	45	45	45	45
8	45	45	45	45	45	45	45	45	45	45	45	45	45
13	45	45	45	45	45	45	45	45	45	45	45	45	45
18	45	45	45	45	45	45	45	45	45	45	45	45	45
23	45	45	45	45	45	45	45	45	45	45	45	45	45

[1] Cut-offs for Description were calculated with the method by Crawford & Howell (1998b). Since no demographic variable was significant in the regression analysis, there is just a single cut-off for all combinations of age and education.

Supplementary Table 3.3. Cut-offs for the Narratives Task.

Narratives – cut-offs

Age

**Education**	20	25	30	35	40	45	50	55	60	65	70	75	80
5	45	45	45	45	45	45	45	45	45	45	45	45	45
8	47	47	47	47	47	47	47	47	47	47	47	47	47
13	49	49	49	49	49	49	49	49	49	49	49	49	49
18	49	49	49	49	49	49	49	49	49	49	49	49	49
23	48	48	48	48	48	48	48	48	48	48	48	48	48

Supplementary Table 3.4. Cut-offs for the Figurative Language 1 Task.

Figurative Language 1 – cut-offs

Age

**Education**	20	25	30	35	40	45	50	55	60	65	70	75	80
5	13	13	13	13	13	13	13	12	12	12	12	12	12
8	13	13	13	13	13	13	13	13	13	12	12	12	12
13	13	13	13	13	13	13	13	13	13	13	13	13	13
18	14	14	14	14	13	13	13	13	13	13	13	13	13
23	14	14	14	14	14	14	14	13	13	13	13	13	13

Supplementary Table 3.5. Cut-offs for the Humor Task.

Humor – cut-offs

Age

**Education**	20	25	30	35	40	45	50	55	60	65	70	75	80
5	4	4	4	4	4	4	4	4	4	3	3	3	3
8	4	4	5	5	5	5	5	4	4	4	4	4	3
13	5	5	5	5	5	5	5	5	5	5	5	4	4
18	5	5	5	5	5	5	5	5	5	5	5	4	4
23	5	5	5	5	5	5	5	5	4	4	4	4	4

Supplementary Table 3.6. Cut-offs for the Figurative Language 2 Task.

Figurative Language 2 – cut-offs

Age

**Education**	20	25	30	35	40	45	50	55	60	65	70	75	80
5	22	22	22	22	21	21	21	20	20	20	20	19	19
8	23	23	22	22	22	22	21	21	21	21	20	20	20
13	24	24	24	23	23	23	22	22	22	22	21	21	21
18	25	25	25	24	24	24	24	23	23	23	22	22	22
23	26	26	26	25	25	25	25	24	24	24	23	23	23

Supplementary Table 3.7. Cut-offs for the Pragmatic production score.

Pragmatic Production – cut-offs

Age

**Education**	20	25	30	35	40	45	50	55	60	65	70	75	80
5	0.96	0.96	0.96	0.96	0.96	0.96	0.95	0.95	0.95	0.95	0.95	0.95	0.95
8	0.96	0.96	0.96	0.96	0.96	0.96	0.95	0.95	0.95	0.95	0.95	0.95	0.95
13	0.96	0.96	0.96	0.96	0.96	0.96	0.95	0.95	0.95	0.95	0.95	0.95	0.95
18	0.96	0.96	0.96	0.96	0.96	0.96	0.95	0.95	0.95	0.95	0.95	0.95	0.95
23	0.96	0.96	0.96	0.96	0.96	0.96	0.95	0.95	0.95	0.95	0.95	0.95	0.95

Supplementary Table 3.8. Cut-offs for the Pragmatic comprehension score.

Pragmatic comprehension – cut-offs

Age

**Education**	20	25	30	35	40	45	50	55	60	65	70	75	80
5	0.76	0.76	0.76	0.76	0.76	0.75	0.75	0.74	0.73	0.72	0.71	0.69	0.67
8	0.81	0.81	0.81	0.81	0.81	0.81	0.8	0.79	0.78	0.77	0.76	0.74	0.73
13	0.86	0.87	0.87	0.87	0.87	0.86	0.86	0.85	0.84	0.83	0.81	0.8	0.78
18	0.88	0.88	0.88	0.88	0.88	0.87	0.87	0.86	0.85	0.84	0.82	0.81	0.79
23	0.84	0.85	0.85	0.85	0.85	0.84	0.84	0.83	0.82	0.81	0.79	0.78	0.76

Supplementary Table 3.9. Cut-offs for the APACS total score.

APACS Total – cut-offs

Age

**Education**	20	25	30	35	40	45	50	55	60	65	70	75	80
5	0.87	0.87	0.87	0.87	0.87	0.86	0.86	0.85	0.85	0.84	0.83	0.83	0.82
8	0.89	0.89	0.89	0.89	0.89	0.89	0.89	0.88	0.88	0.87	0.86	0.85	0.84
13	0.92	0.92	0.92	0.92	0.92	0.92	0.91	0.91	0.9	0.9	0.89	0.88	0.87
18	0.92	0.92	0.93	0.92	0.92	0.92	0.92	0.91	0.91	0.9	0.89	0.88	0.87
23	0.9	0.9	0.91	0.9	0.9	0.9	0.9	0.89	0.89	0.88	0.87	0.86	0.85
